# Supplementary material for: Biofilm Production by Enterotoxigenic Strains of Bacillus cereus in Different Materials and under Different Environmental Conditions
Source: Microorganisms. 2020 Jul 17;8(7):1071. doi: 10.3390/microorganisms8071071 (PMC7409032; doi:10.3390/microorganisms8071071)
Supplement: Supplementary file 1 [file microorganisms-08-01071-s001.pdf]

Supplementary material (S1)

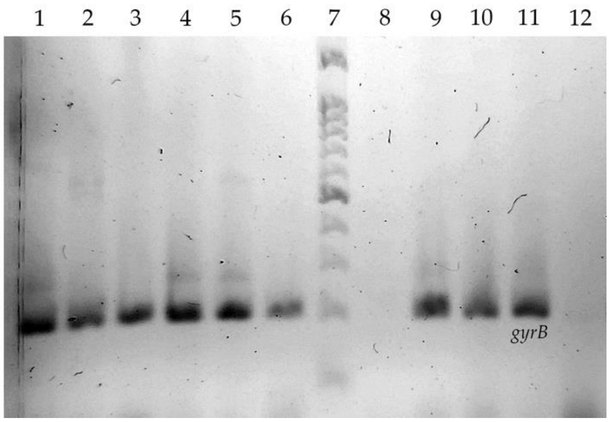

Figure 1. *gyrB* gene amplification. 1. B013, 2. B030, 3. B092, 4. B093, 5. B171, 6. B186, 7. Molecular weight marker, 8. B027, 9. B362, 10. B326 11. ATCC 14579, 12. B067.

Supplementary material (S2)

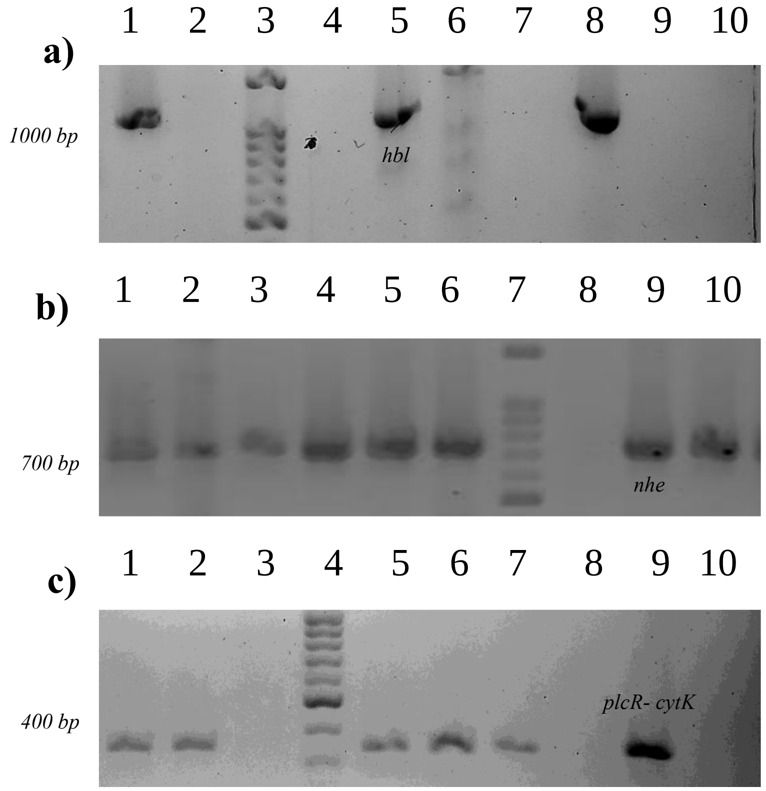

Figure 2. Enteroxigenic profile. a) *hlb*, 1. ATCC 14579, 2. B013, 3. Molecular weight marker, 4. B092, 5. B030, 6. B093, 7. B171, 8. B326, 9. B362 10. B186. **b)** *nhe,* 1.B013, 2.B030, 3. B092, 4. B093, 5. B171, 6. B186, 7. Molecular weight marker, 8. ATCC 14579, 9. B362, 10. B326. **c)** *plcR- cytK*, 1. ATCC 14579, 2. B013, 3. B092, 4. Molecular weight marker, 5. B030, 6. B171, 7. B186, 8. B093, 9. B326, 10. B362.
